# Danggui Buxue Tang Inhibits 2,4-Dinitrochlorobenzene: Induced Atopic Dermatitis in Mice

**DOI:** 10.1155/2015/672891

**Published:** 2015-03-10

**Authors:** Li-Wen Fang, Chao-Chun Cheng, Tzann-Shun Hwang, Wen-Chung Huang, Chian-Jiun Liou, Wen-Chyuan Chen, Shu-Ju Wu

**Affiliations:** ^1^Department of Nutrition, I-Shou University, Yanchao District, Kaohsiung City, Taiwan; ^2^Department of Nutrition and Health Sciences, Chang Gung University of Science and Technology, Guishan District, Taoyuan City, Taiwan; ^3^Nutritional Science Department, Fu Jen Catholic University, New Taipei City, Taiwan; ^4^Graduate Institute of Biotechnology, Chinese Culture University, Yang-Ming-Shan, Taipei, Taiwan; ^5^Graduate Institute of Health Industry Technology, Chang Gung University of Science and Technology, Guishan District, Taoyuan City, Taiwan; ^6^Research Center for Industry of Human Ecology, Chang Gung University of Science and Technology, Guishan District, Taoyuan City, Taiwan; ^7^Department of Nursing, Chang Gung University of Science and Technology, Guishan District, Taoyuan City, Taiwan; ^8^Center for General Education, Chang Gung University of Science and Technology, Guishan District, Taoyuan City, Taiwan

## Abstract

Danggui Buxue Tang (DBT) is a herbal decoction that has been used in Chinese medicine to enhance qi and blood circulation. Previously, we found that DBT can suppress allergy-related asthma in mice, leading us to hypothesize that DBT might ameliorate allergy disease. In this study, we evaluated whether DBT can attenuate atopic dermatitis (AD) symptoms and have an anti-inflammatory effect on AD-like mice. The dorsal skin of female mice was shaved and sensitized cutaneously (skin smear) with 1-chloro-2,4-dinitrobenzene. Mice were then given various doses of DBT from days 14 to 29 cutaneously. DBT treatment suppressed ear swelling and skin inflammation and decreased mast cell and eosinophil infiltration into skin and ear tissue. DBT also inhibited levels of IgE and Th2-associated cytokine levels in serum. These results demonstrate that cutaneous administration of DBT reduced the development of AD-like skin lesions in mice.

## 1. Introduction

Atopic dermatitis (AD) is a chronic inflammatory allergic and relapsing skin disease, and its morbidity has been increasing gradually in developing and developed countries [1]. Approximately 50% of patients experience onset before the age of 5 years [2]. AD attacks are characterized by redness and itching in the responsive skin, and excessive scratching can cause skin cracking and fluid leakage [3]. In patients with chronic AD, the skin will gradually thicken and become rough, affecting its appearance. These pathological characteristics can interfere with mood and lifestyle [4].

Treatment of AD consists mainly of steroid cream applied to the skin [5]. However, steroids are immunosuppressants and do not reduce Th2 cell function, although they do suppress immunity and increase the risk of bacterial infection [6]. Furthermore, long-term topically applied steroids also lead to thinning of the skin and to cracking and bleeding [7].

Previous studies found that activation of T cells, especially Th2 cells, can lead to induction of the allergic response [8]. Th2 cells secrete IL-4 to activate B cells for IgE production, thus inducing activation of mast cells and causing the allergic reaction [9]. In addition, Th2 cells secrete IL-5 to induce eosinophil differentiation and infiltration into the allergic skin tissue [10]. Thus, inhibiting the activity of Th2 cells may improve skin symptoms of AD.

Danggui Buxue Tang (DBT) is often used in Chinese medicine in China and Taiwan [11]. It consists of* Angelica sinensis* and* Astragalus membranaceus* (1 : 5) and is predominately used to increase blood circulation and qi [12]. Recent studies have found that DBT improves fibrosis of the lung in rat and also decreases angiogenesis and oxidative stress in rat liver fibrosis [13, 14]. Another group found that DBT could modulate hematopoietic function [15], and human trials have revealed that DBT enhances quality of life for postmenopausal women by decreasing hot flashes and night sweats [16]. Our previous study found that DBT significantly suppresses airway hyperresponsiveness and eosinophil infiltration in mice by blocking Th2 cytokine production. Because AD is a disease of excessive Th2 cytokine production [11], we evaluated whether DBT has a therapeutic effect by suppressing this response in AD-like skin lesions in mice.

## 2. Materials and Methods

### 2.1. Animals

All animal experimental protocols were approved by the Animal Care Committee of Chang Gung University of Science and Technology and Chang Gung University (IACUC approval number: 2012-001). Eight-week-old female BALB/c mice were purchased from the National Laboratory Animal Center (Taiwan) and housed at a consistent temperature (23 ± 2°C) in the air-controlled conventional animal room at the Animal Center of Chang Gung University.

### 2.2. Preparation of DBT

Danggui Buxue Tang (DBT), which contains* Astragalus membranaceus* (AM) and* Angelica sinensis* (AS) (AM : AS = 5 : 1), was prepared as previously described [11]. In brief, AS and AM were planted and collected from Gansu and Shanxi province, respectively, China. The roots of 100 g AS and AM were soaked in 1000 mL water and boiled for 60 minutes, respectively. Then, extracts were centrifuged, and the supernatants were lyophilized. AS and AM were mixed with excipients. The powder contained 1.1 g/g AS or 1.4 g/g AM, respectively.

### 2.3. Sensitization, Challenge, and Drug Treatment

**Figure 1:**
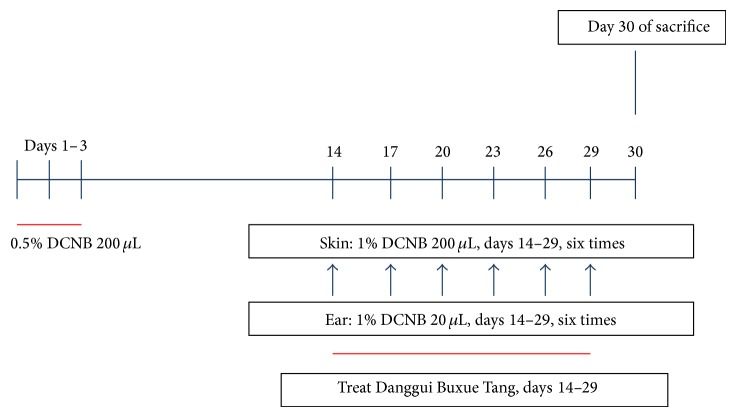
A summary of the development of AD-like skin lesions in DNCB-treated mice. The dorsal skin of mice was shaved and treated with topical 0.5% DNCB on days 1–3. Next, the mice were challenged with 1% DNCB on days 14, 17, 20, 23, 26, and 29. AD-like mice were treated with topical Danggui Buxue Tang (DBT) on days 14–27. Finally, mice were sacrificed on day 30.

The dorsal skin of the mice was shaved and sensitized by application to the skin of 0.5% 1-chloro-2,4-dinitrobenzene (DNCB, Sigma-Aldrich, St. Louis, MO, USA) as previously described [17]. Briefly, BALB/c mice were shaved of dorsal hair and 200 *μ*L 0.5% DNCB in acetone/olive oil (3 : 1) was applied to the shaved area on experimental days 1–3 (Figure 1). On days 14–29, mice were challenged with 100 *μ*L 1% DNCB on each ear and on the dorsal skin on days 14, 17, 20, 23, 26, and 29.

Mice were randomly divided into four groups (*n* = 8 per group): normal control mice (N group) were sensitized and challenged with normal saline; sensitized control mice (S group) were sensitized and challenged with DNCB; and experimental mice were sensitized and challenged with DNCB and topical skin treatment with 3 g/kg or 10 g/kg DBT (groups designated as HD3 and HD10, resp.). All drug treatments were applied to the backs of the mice from days 14 to 29 as described in Figure 1.

### 2.4. Measurement of Ear Thickness

On day 30, ear thickness was measured using a dial gauge (Mitutoyo, Tokyo, Japan) as previously described [17].

### 2.5. Histopathological Studies

The ears and dorsal skin were fixed in 10% formalin. The tissues were cut into 6 *μ*m thick sections and stained with hematoxylin and eosin (H&E) and toluidine blue as previously described [17]. The sections were observed using a light microscope at 100–200x magnification, and the mast cells were measured under 10–15 high-power fields (HPFs).

### 2.6. Serum Collection and Splenocyte Cultures

Blood was collected and centrifuged to harvest serum. Serum and spleen cells were isolated as previously described [11], and cells (5 × 10^6^ cells/mL) were then stimulated with 2 *μ*g/mL concanavalin A (Con A) for 2 days in RPMI 1640 medium. The supernatants were collected and cytokine levels measured by ELISA.

### 2.7. Measurement of Cytokine and Antibody Levels

Cytokines and antibodies were measured as previously described [18]. Supernatants of spleen cells were measured for the specific cytokines, including interferon (IFN-*γ*), IL-4, IL-5, and tumor necrosis factor (TNF-*α*) (R&D Systems, Minneapolis, MN, USA), as were levels of serum antibodies IgG1, IgG2a, and IgE (BD Biosciences, San Diego, CA, USA) by ELISA kits.

### 2.8. Statistical Analysis

All statistical significance was determined by one-way ANOVA, followed by Dunnett's post hoc test. Results are presented as mean ± SEM (standard error of the mean), and *P* values were considered statistically significant at <0.05.

## 3. Results

### 3.1. DBT Inhibits Ear Swelling and Ameliorates AD Symptoms in AD-Like Mice

**Figure 2:**
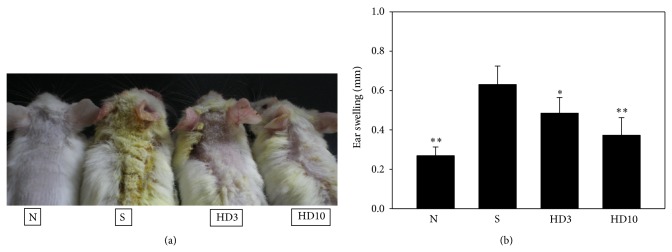
Clinical features of AD-like skin lesions treated topically with DBT. (a) Normal control mice (N); sensitized control mice (S); sensitized mice topically treated with 3 g/kg DBT (HD3) or 10 g/kg DBT (HD10). Ear thicknesses were also calculated (b). *n* = 8 mice per group for all groups. Data are presented as means ± SEM. ^*^ *P* < 0.05, ^**^ *P* < 0.01 versus sensitized control mice.

DNCB-sensitized mice clearly manifested AD-like syndromes in the skin and ear, including edema, erythema, scarring, and excoriation compared with normal mice (Figure 2(a)). Because DNCB could induce ear swelling in this experimental model, we measured ear thickness on day 30 (Figure 2(b)). Different topical doses of DBT to DNCB-sensitized mice significantly inhibited ear swelling compared with DNCB-sensitized mice (HD3: 0.49 ± 0.09 mm, *P* < 0.05; HD10: 0.37 ± 0.08 mm, *P* < 0.01, vs. the S group: 0.63 ± 0.05 mm).

### 3.2. DBT Decreases Eosinophil and Mast Cell Infiltration in AD-Like Mice

**Figure 3:**
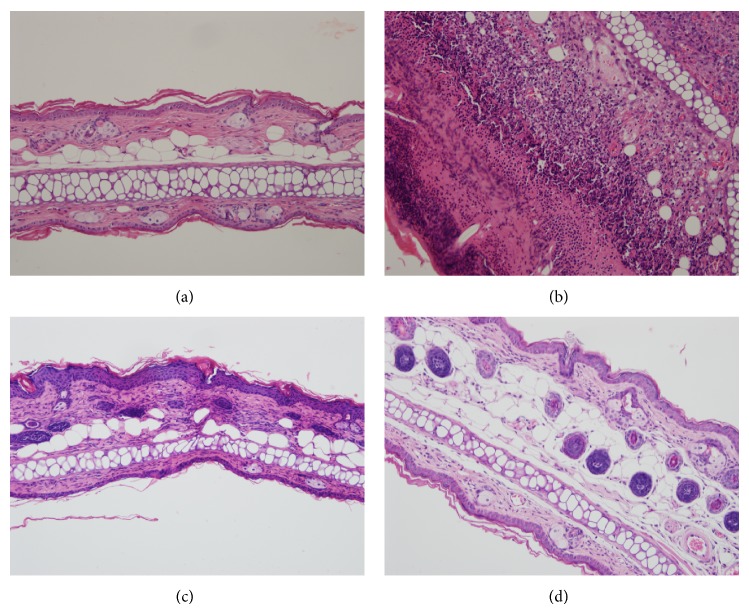
The effects of DBT on eosinophil infiltration in the ears. Ear sections were stained with H&E and analyzed for eosinophil infiltration in normal control mice (a); sensitized control mice (b); 3 g/kg DBT (HD3, c) or 10 g/kg DBT (HD10, d) (100x magnification); *n* = 8 mice per group for all groups.

**Figure 4:**
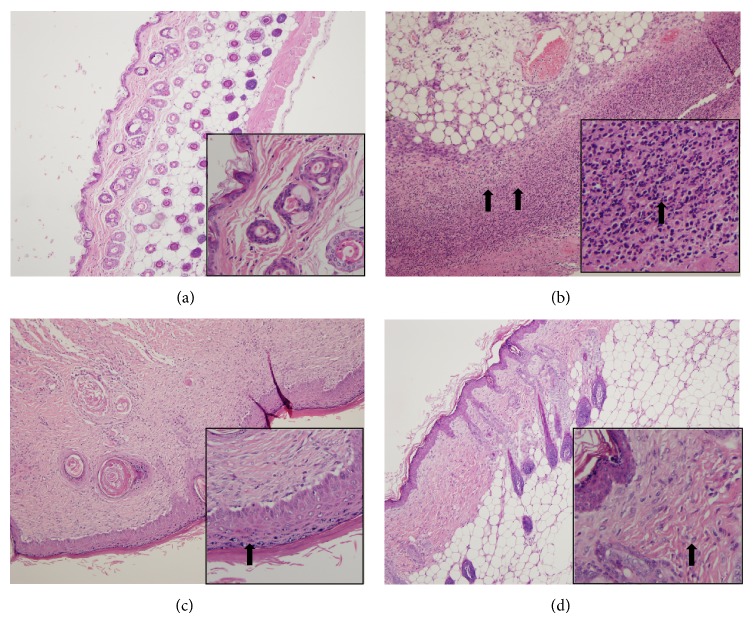
The effects of DBT on eosinophil infiltration in skin. Skin sections were stained with H&E and analyzed for eosinophil infiltration in normal control mice (a); sensitized control mice (b); 3 g/kg DBT (HD3, c) or 10 g/kg DBT (HD10, d) (100x magnification, and amplified graph is 200x); *n* = 8 mice per group for all groups. Black arrows indicate eosinophils.

**Figure 5:**
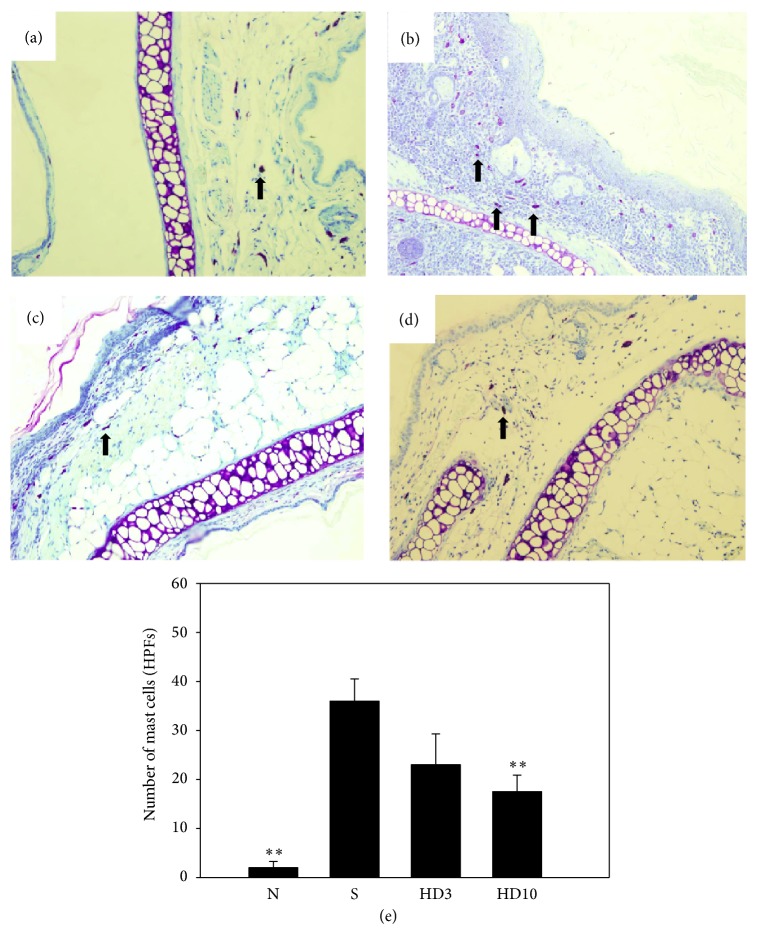
The effects of DBT on mast cell infiltration in ear tissue. Ear sections were stained with toluidine blue to analyze mast cell infiltration in normal control mice (a); sensitized control mice (b); 3 g/kg DBT (HD3, c) or 10 g/kg DBT (HD10, d) (100x magnification); *n* = 8 mice per group for all groups. Arrows indicate mast cells. The mast cells were measured under 10–15 high-power fields (HPFs) (e). Data are presented as means ± SEM. ^*^ *P* < 0.05, ^**^ *P* < 0.01 versus sensitized control mice.

**Figure 6:**
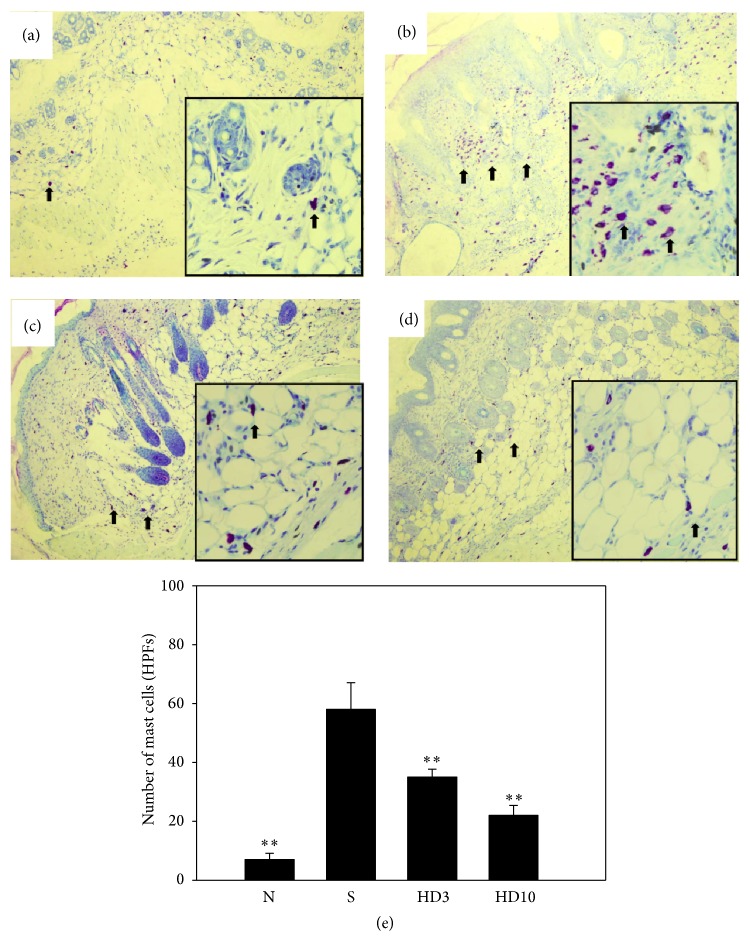
The effects of DBT on mast cell infiltration in skin tissue. Skin sections were stained with toluidine blue to analyze mast cell infiltration in normal control mice (a); sensitized control mice (b); 3 g/kg DBT (HD3, c) or 10 g/kg DBT (HD10, d) (100x magnification, and amplified graph is 200x); *n* = 8 mice per group for all groups. Arrows indicate mast cells. The mast cells were measured under 10–15 high-power fields (HPFs) (e). Data are presented as means ± SEM. ^*^ *P* < 0.05, ^**^ *P* < 0.01 versus sensitized control mice.

To investigate the effect of DBT in AD-like skin lesions, ear and skin sections were stained with H&E to examine epidermal thickness and eosinophil infiltration (Figures 3 and 4). In brief, DNCB-sensitized mice exhibited thickening of the epidermal layer and more eosinophil infiltration compared with normal mice. Topical administration of DBT significantly decreased the epidermal layer and eosinophil infiltration compared with DNCB-sensitized mice. In addition, topical administration of DBT also suppressed mast cell infiltration in the ears and skin compared with DNCB-sensitized mice (ear: HD3: 22.5 ± 6.8, *P* = 0.14; HD10: 18.5 ± 2.4, *P* < 0.01, vs. the S group: 36.0 ± 4.5, Figure 5; and skin: HD3: 33.6 ± 4.1, *P* < 0.01; HD10: 22.8.5 ± 5.6, *P* < 0.01, vs. the S group: 61.4 ± 5.7, Figure 6).

### 3.3. DBT Affects Serum Antibodies and Cytokines

**Figure 7:**
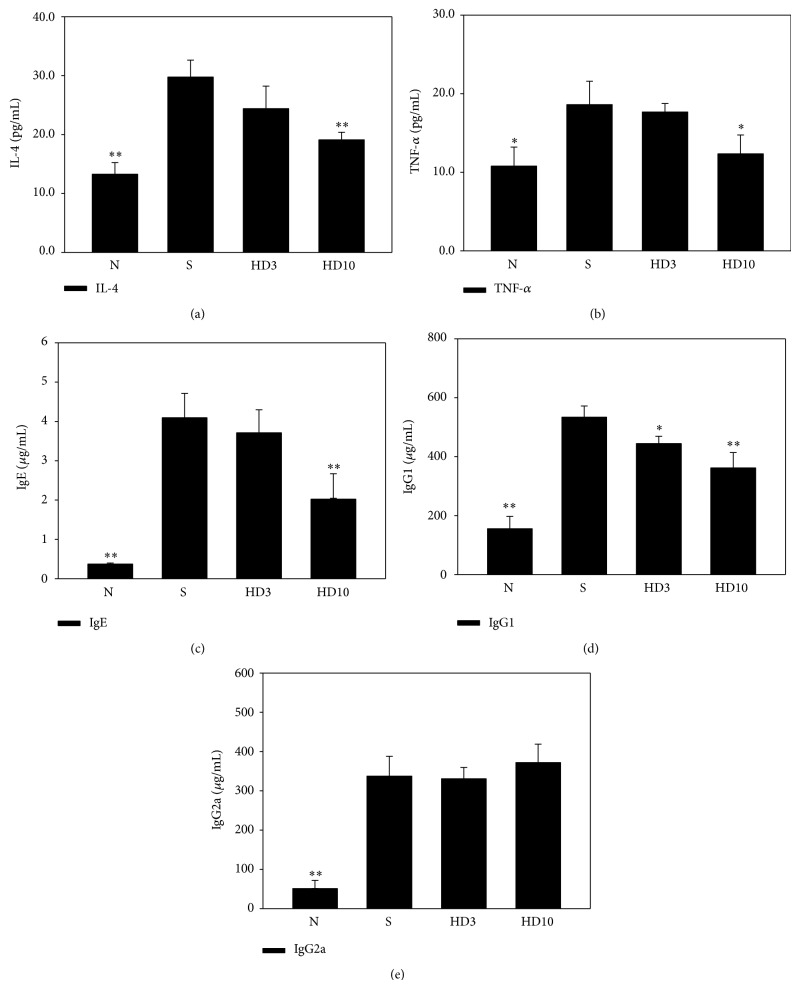
Effect of DBT on levels of serum IL-4 (a), TNF-*α* (b), serum antibodies IgE (c), IgG1 (d), and IgG2a (e). Serum was centrifuged and collected; then the cytokine and antibody levels were evaluated by ELISA. Data are presented as means ± SEM. ^*^ *P* < 0.05, ^**^ *P* < 0.01 versus sensitized control mice.

Next, we examined antibody and cytokine levels to evaluate whether DBT could modulate the allergic or inflammatory response in serum (Figure 7). Topical administration of DBT significantly decreased IL-4 and TNF-*α* levels compared with DNCB-sensitized mice (IL-4, HD3: 25.4 ± 2.4 pg/mL, *P* = 0.23; HD10: 18.3 ± 2.1 pg/mL, *P* < 0.01, vs. S: 29.8 ± 2.9 pg/mL, resp.; and TNF-*α*, HD3: 17.3 ± 2.1 pg/mL, *P* = 0.18; HD10: 13.2 ± 1.5 pg/mL, *P* < 0.05, vs. S: 19.5 ± 2.1 pg/mL, resp.). In addition, DBT also significantly suppressed IgE and IgG1 production but did not modulate the IgG2a level.

### 3.4. DBT Suppresses Th2-Associated Cytokines in Spleen Cell Cultures

**Figure 8:**
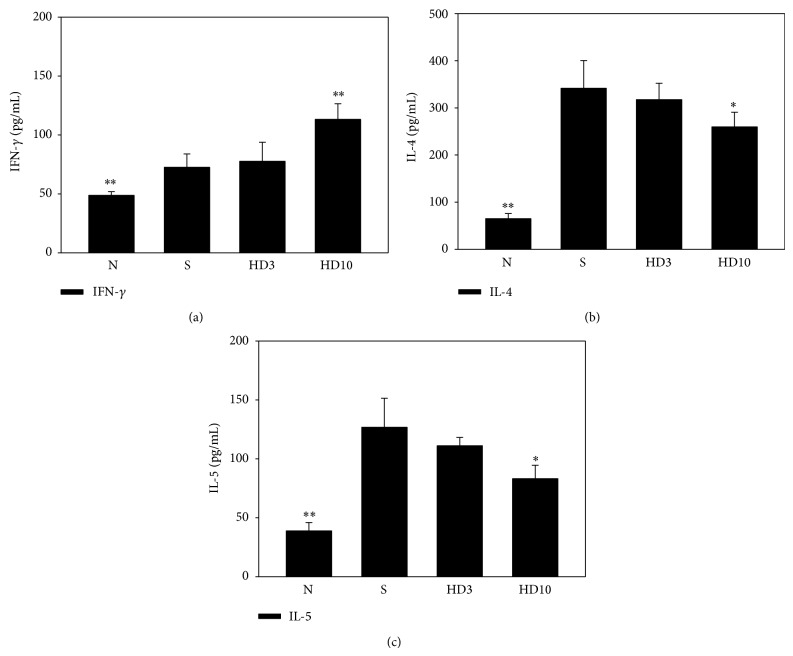
Effect of DBT on cytokine levels of spleen cells. The cells were stimulated with 2 *μ*g/mL concanavalin A (Con A) for 2 days. The supernatants were collected and assayed the levels of IFN-*γ* (a), IL-4 (b), and IL-5 (c). Data are presented as means ± SEM. ^*^ *P* < 0.05, ^**^ *P* < 0.01 versus sensitized control mice.

Splenocytes were isolated and stimulated with Con A for 2 days. Topical administration of 10 g/kg DBT decreased the levels of IL-4 and IL-5 and increased IFN-*γ* production compared with DNCB-sensitized mice (Figure 8).

## 4. Discussion

AD is a chronic inflammatory skin disease, and skin lesions are characterized by erythema, eczema, and itching [4]. The repeated allergic, inflammatory skin response and the remodeling of the skin surface can lead to hardening and thickening of the skin [19]. In addition, intense pruritus can interfere with work and sleep, and roughening, edema, and hemorrhage of skin also affect its appearance [20].

Topical steroids are used to treat AD clinically, and antihistamine and antibiotic ointments often also are used to improve the chronic itching and edema and prevent bacterial infection [21]. However, the long-term use of steroids may involve side effects and cause skin thinning and brittleness or even skin cracking [22]. Previous studies found that* Staphylococcus aureus* has been isolated in the inflamed skin of AD patients.* S. aureus* can release a superantigen that induces more T cell activity and aggravates skin inflammation and allergy symptoms [23]. Hence, AD patients need to use topical antibiotics to inhibit bacterial colonization and decrease skin inflammation. Recent studies have found that* S. aureus* has tolerance to steroids and antibiotics [5]. Therefore, in recent years, many researchers have sought other therapies to address AD.

Traditional Chinese medicine has used acupuncture and Chinese herbal therapies to treat AD for thousands of years [24–26]. Chinese herbal medicine has a basis in accumulated clinical experience and has resulted in several herbal formulas to treat AD, including Xiao Feng San, Xiaofeng Daochi Tang, Longdan Xiegan Tang, and Yangxue Dingfeng Tang [5, 8, 27]. Previous study has found that oral administration of Xiao Feng San significantly suppresses desquamation and lichenification of the skin [28]. Another study has shown that a formula containing* Flos Lonicerae* (Jin Yin Hua),* Herba Menthae* (Bo He),* Cortex Moutan* (Dan Pi),* Rhizoma Atractylodis* (Cang Zhu), and* Cortex Phellodendri* (Huang Bai) and used in clinical trials in children with AD can improve quality of life and reduce topical steroid use in moderate to severe AD patients [27]. In addition, another herb has been reported that could improve symptoms in AD-like mice [29–31].

Previous studies have reported that DBT can reduce bleomycin-induced pulmonary fibrosis in rats by boosting matrix metalloproteinases, MMP-1, MMP-9, and TIMP-1 expression [14, 23]. DBT treatment in ovariectomized rats modulates levels of follicle-stimulating hormone and luteinizing hormone in serum, and it may improve estrogen levels in women after menopause [32]. Previously, we found that DBT could improve asthma symptoms in asthmatic mice, including attenuating eosinophil infiltration and airway hyperresponsiveness and decreasing Th2 cytokine production in bronchoalveolar lavage fluid [11]. In this study, we confirmed that DBT could reduce ear swelling in AD-like mice and improve skin redness and scaling and bleeding symptoms. The herbal formula also reduced eosinophil and mast cell infiltration in the skin and ear, suppressed IgE levels in serum, and improved Th2 cytokine in serum and spleen cells.

Previous findings have suggested that Th2 cells modulate IgE-dependent mast cell activation and degranulation in allergic AD [9, 33]. More evidence has shown that Th2 cells secreting excessive IL-4 will exacerbate the symptoms of AD [8]. IL-4 predominately induces B cell activation and releases more IgE to bind mast cells. Mast cells reside in almost tissues of the body, and they are majorly located at skin, airways, urogenital tracts, and intestinal tracts [9]. Chemokine receptors (CXCR1 and CXCR2) of activated mast cells receive cytokine (CCL5) signals, and stimulated mast cells migrate into the tissue to release histamine, lipid mediators, and other inflammatory cytokines and chemokines to cause serious allergy and inflammation [10]. In patients with atopic dermatitis, the skin will have a lot of mast cell infiltration to cause severe inflammatory skin. Mast cells would decrease the function of connective tissue and damaged the skin barrier [9]. In this experiment, DBT significantly suppressed IgE levels in serum and inhibited mast cell infiltration in the skin and the ear to decrease inflammation and swelling. Hence, we suggest that DBT has an effect on the regulation of cytokine IL-4 expression and mast cell infiltration to improve AD-like syndromes in the skin, including erythema, edema, skin cracking, and fluid leakage.

In atopic dermatitis, Th2 cells were leading to excessive IL-5 productions [8]. IL-5 stimulates bone marrow cells to produce more eosinophils, and the surfaces of differentiated eosinophils express CCR3, which binds to chemokines CCL11 and CCL24 to induce eosinophil migration and infiltration into the skin [34]. These activated eosinophils can release more eosinophil peroxidases and leukotrienes to cause skin inflammation. DBT did not promote or decrease CCL11 in serum (data not shown). Thus, it was an important finding that DBT attenuates IL-5 production in reducing eosinophil activation and migration into the skin in a mouse model of AD.

Proinflammatory cytokines also affect swelling and inflammation in AD-like skin [8]. We sought to extract mouse skin proteins and detect IL-6 and TNF-*α* by ELISA. However, those proinflammatory cytokines were not detected in our system (data not shown). Hence, we assayed serum and found that DBT could decrease TNF-*α* production to ameliorate the inflammatory response. In this experiment, we also found that levels of IFN-*γ* also increased in spleen cell culture and that TNF-*α* and IFN-*γ* were mainly secreted by Th1 cells. Previous findings have suggested that atopic dermatitis is mainly Th1/Th2 imbalance [8, 10]. Dendritic cells of skin captured allergy to induce the overexpression of Th2 cells to secrete more the levels of IL-4 and IL-5. Those Th2 cytokines induce the infiltration of eosinophils and mast cells to release inflammatory mediators [10, 22]. Therefore, DBT inhibited the activity of Th2 cells, and Th1 cells also had a moderating effect in the AD-like mice. DBT was formed by* Angelica sinensis *and* Astragalus membranaceus*. Recent studies have found that topical application of* Astragalus membranaceus *also improve the symptom of AD in DNCB-induced mice [35]. We would investigate which compound of DBT is main effect to improve the symptom of AD in the future.

## 5. Conclusion

In conclusion, DBT reduces inflammatory symptoms in AD-like mice by suppressing the production of Th2-associated and proinflammatory cytokines to suppress eosinophil and mast cell infiltration.
